# Liquid biopsy using ascitic fluid and pleural effusion supernatants for genomic profiling in gastrointestinal and lung cancers

**DOI:** 10.1186/s12885-022-09922-5

**Published:** 2022-09-27

**Authors:** Huita Wu, Haonan Ji, Wenhui Yang, Min Zhang, Yifang Guo, Bangkai Li, Jiayin Wang, Rongrong Chen, Yuan Chen, Xin Wang

**Affiliations:** 1 Department of Oncology, Zhongshan Hospital, Xiamen University, Xiamen, 361004 Fujian China; 2 Fujian Provincial Key Laboratory of Chronic Liver Disease and Hepatocellular Carcinoma, Zhongshan Hospital, Xiamen University, Xiamen, 361004 Fujian China; 3 Department of Clinical Medicine, Fujian Medical University, Fuzhou, 350122 Fujian China; 4 Shanxi Bethune Hospital, Taiyuan, China; 5 School of Computer Science and Technology, Xi’an Jiaotong University, Xi’an, China; 6 Geneplus-Beijing, Beijing, China; 7 Department of Oncology and Hematology, Xiamen Haicang Hospital, Xiamen, 361026 Fujian China; 8 Emergency Department (Outpatient Chemotherapy Center), The Third Affiliated Hospital of Kunming Medical University/Yunnan Cancer Hospital, Kunming, 650118 Yunnan China; 9 Department of Oncology, Tongji Hospital, Tongji Medical College, Huazhong University of Science and Technology, Wuhan, 430030 Hubei China

**Keywords:** Ascites, Pleural effusion, Next generation sequencing, Peritoneal metastasis, Pleural metastasis

## Abstract

**Background:**

Precision medicine highlights the importance of incorporating molecular genetic testing into standard clinical care. Next-generation sequencing can detect cancer-specific gene mutations, and molecular-targeted drugs can be designed to be effective for one or more specific gene mutations. For patients with special site metastases, it is particularly important to use appropriate samples for genetic profiling. This study aimed to determine whether genomic profiling using ASC and PE is effective in detecting genetic mutations.

**Methods:**

Tissues, plasma, ascites (ASC) supernatants, and pleural effusion (PE) samples from gastrointestinal cancer patients with peritoneal metastasis and lung cancer patients with pleural metastasis were collected for comprehensive genomic profiling. The samples were subjected to next-generation sequencing using a panel of 59 or 1021 cancer-relevant genes panel.

**Results:**

A total of 156 tissues, 188 plasma samples, 45 ASC supernatants, and 1 PE samples from 304 gastrointestinal cancer patients and 446 PE supernatants, 122 tissues, 389 plasma samples, and 45 PE sediments from 407 lung cancer patients were analyzed. The MSAF was significantly higher in ASC and PE supernatant than that in plasma ctDNA (50.00% vs. 3.00%, *p* < 0.0001 and 28.5% vs. 1.30%, *p* < 0.0001, respectively). The ASC supernatant had a higher actionable mutation rate and more actionable alterations than the plasma ctDNA in 26 paired samples. The PE supernatant had a higher total actionable mutation rate than plasma (80.3% vs. 48.4%, *p* < 0.05). The PE supernatant had a higher frequency of uncommon variations than the plasma regardless of distant organ metastasis.

**Conclusion:**

ASC and PE supernatants could be better alternative samples when tumor tissues are not available, especially in patients with only peritoneal or pleural metastases.

**Supplementary Information:**

The online version contains supplementary material available at 10.1186/s12885-022-09922-5.

## Background

Precision medicine is designed to treat individual patients with the most suitable therapy at the most appropriate time based on the patient’s biological and molecular features. This highlights the importance of incorporating molecular genetic testing into standard clinical care. Next-generation sequencing (NGS) can detect cancer-specific gene mutations, and molecular-targeted drugs can be designed to be effective for one or more specific gene mutations. However, molecular profiling of tumor tissues from advanced patients is not always possible. Thus, tumor-derived cell-free DNA (cfDNA) isolated from body fluids, including plasma, pleural effusions (PE), cerebrospinal fluids, saliva, and urine, is being investigated in cancer genomic profiling [1–4].

Gastrointestinal (GI) and lung cancers have a high incidence and mortality rate worldwide. Peritoneal metastasis appears to be a common pattern and is associated with a poorer prognosis than other sites in gastric (GC) and colorectal cancers (CRC) [5, 6]. The pleura is also a common metastatic site in lung cancer and worsens the survival rate [7]. Ascites (ASC) and PE are usually available in large quantities, and can be extracted using minimally invasive procedures in patients with peritoneal and pleural involvement. ASC and PE contain floating malignant cells as well as tumor cfDNA in the supernatant, and the cytological and supernatants have been used to detect genetic mutations using NGS in multiple types of tumors [8, 9]. However, the performance of genomic profiling using ASC and PE in the real-world settings has not yet been fully investigated.

In this retrospective study, we collected ASC, plasma, tissue, and PE samples from GI cancer patients with who had peritoneal metastasis as well as and plasma, tissue, and PE samples from lung cancer patients with pleural metastasis to verify the efficacy of ASC and PE in detecting genetic mutations. Paired samples from a subset of patients were compared to systematically evaluate the concordance of the genomic profiles from different sample types.

## Materials and Methods

### Patients and samples

In the GI cohort, 304 patients with peritoneal metastasis were retrospectively enrolled. A total of 390 samples, including 156 tissues, 188 plasma samples, 45 ASC supernatants, and 1 PE sample, were used to analyze the applicability of ascitic fluid in detecting genetic mutations via NGS.

A total of 407 patients with pleural metastasis were retrospectively enrolled in the lung cancer cohort. A total of 1002 samples, including 446 PE supernatants, 122 tissues, 389 plasma samples, and 45 PE sediments, were used to analyze the efficacy of PE in detecting genetic mutations using NGS.

### Next-generation sequencing

ASC and PE were centrifuged at 1600 × g for 10 min and 16,000 × g for 10 min, respectively. Supernatants were used for cfDNA extraction, and sediments were used for genomic DNA extraction. Circulating cell-free DNA (cfDNA) was isolated from the supernatant and plasma using the QIAamp Circulating Nucleic Acid Kit (Qiagen, Valencia, CA, USA). DNA from tumor tissues and sediments was extracted using the QIAamp DNA Mini Kit (Qiagen, Valencia, CA, USA). White blood cell DNA was used as the germline control and was extracted using the DNeasy Blood Kit (Qiagen, Valencia, CA) [10]. Sequencing libraries were prepared from cfDNA using KAPA DNA Library Preparation Kits (Kapa Biosystems, Wilmington, MA, USA), and genomic DNA sequencing libraries were prepared using Illumina TruSeq DNA Library Preparation Kits (Illumina, San Diego, CA, USA). A total of 1392 libraries from 711 patients were hybridized with custom-designed biotinylated oligonucleotide probes (Roche NimbleGen, Madison, WI, USA) on 59 genes or 1021 cancer-related gene panels (Supplementary Table 1) using the Illumina Nextseq CN 500 or Gene + Seq 2000 instrument [11, 12].

Sequencing data were analyzed using the default parameters [13]. The reads with removed adaptor sequences and low-quality reads were aligned to the reference human genome (hg19) using the Burrows-Wheeler Aligner (BWA; version 0.7.12-r1039). Realignment and recalibration were performed by using GATK (version 3.4–46-gbc02625) [14]. Single nucleotide variants (SNV) were called using MuTect (version 1.1.4) and NChot, a software developed in-house to review hotspot variants. Small insertions and deletions (Indel) were determined using GATK. Somatic copy number variations (CNV) were identified using CONTRA (v2.0.8) [15]. The fusion genes were identified with the NCsv program (in-house) using split reads, discordant pair reads, and single unmapped reads in the alignment file. The final candidate variants were all manually verified using the Integrative Genomics Viewer. Targetable genomic alterations simultaneously detected by this assay include SNV, Indel, CNV and fusions. Allele frequency was defined as the number of mutant reads / number of all aligned reads at this site (mutant + wildtype), and MSAF was the maximum somatic allele frequency.

### Statistical analysis

The MSAF in each group did not conform to the normal distribution, and the Mann–Whitney test was used to test the difference in MSAF between the groups. Fisher’s exact test was used to test the difference in mutation detection rates between groups. Statistical significance was defined as a *P*-value of < 0.05. Statistical analyses were performed using the GraphPad Prism 8.0 program.

## Results

### Study design and GI cancer patient characteristics

This study enrolled 304 GI cancer patients with peritoneal metastasis at diagnosis or during disease progression (Table 1). The median age at diagnosis was 57 (range 20–93), and 145 (47.7%) were female. The major cancer type were CRC, and GC, followed by appendiceal cancer, pancreatic cancer, small intestinal and ampullary carcinoma, and esophageal cancer. One hundred and sixty-nine (55.6%) patients had distant organ metastasis, except in the peritoneum. Two hundred and five patients (67.4%) had previous systemic treatment. A total of 390 specimens were collected to analyze the efficacy of mutation detection among different real-world samples, including 188 plasma samples, 156 tissue samples, 45 ASCs supernatant, and 1 PE supernatant.

**Table 1:**
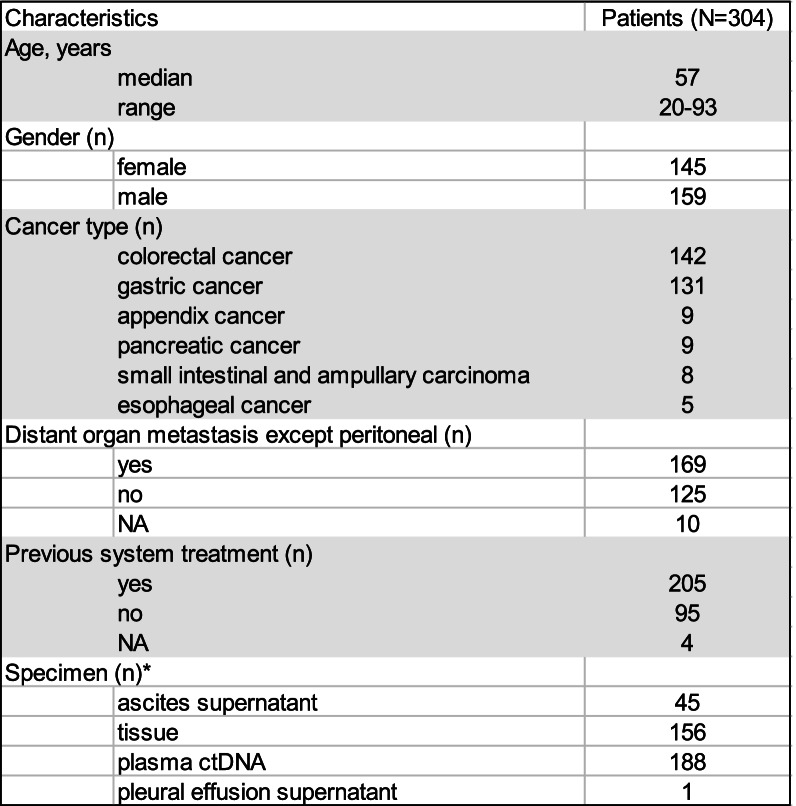
Clinical pathological characteristics of gastrointestinal cancer patients

^*^Including 71 patients who had multiple samples

*Abbreviations: NA* not available, *ctDNA* circulating tumor DNA

### ASC supernatants from real-world samples

**Fig. 1:**
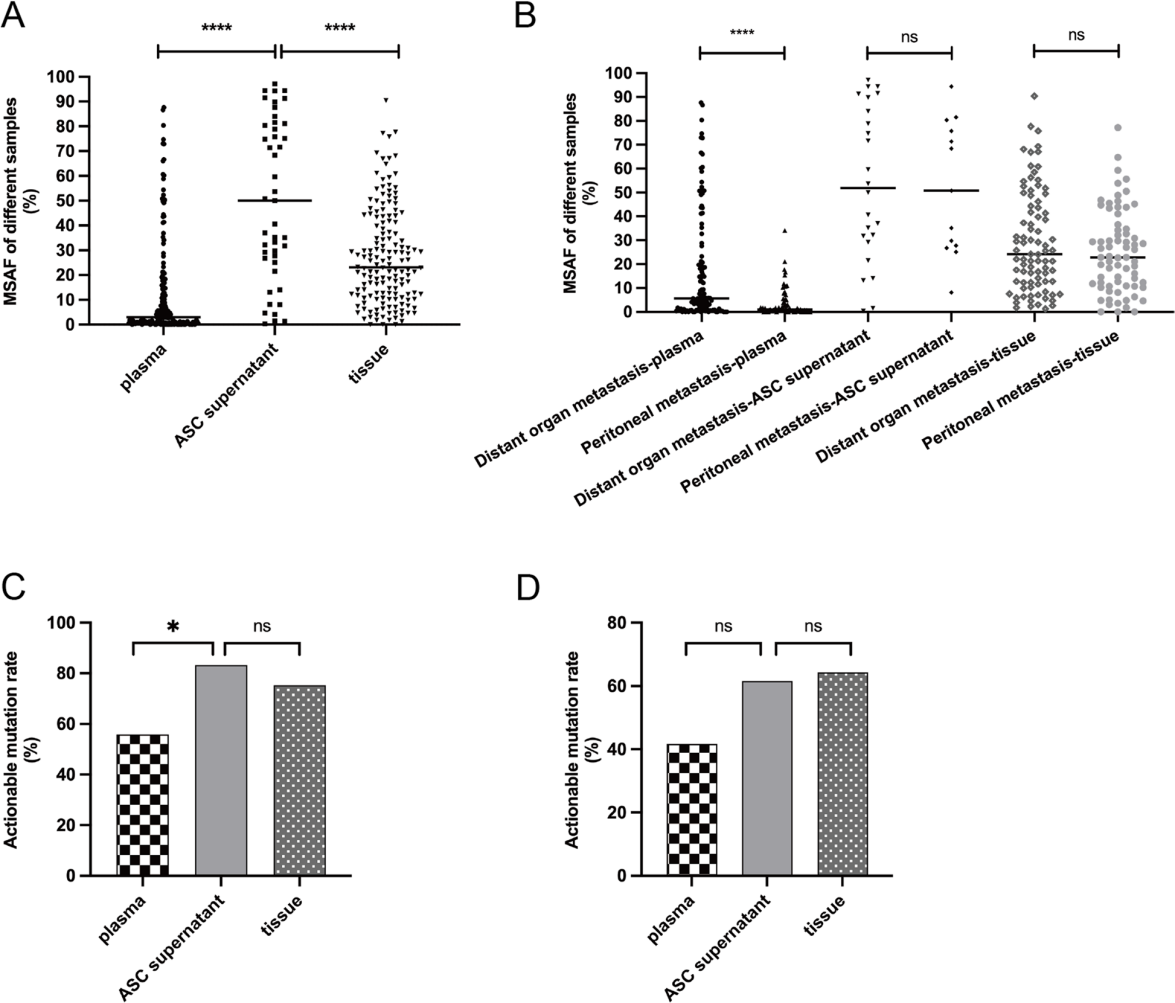
The mutation detection ability of different samples in gastrointestinal cancer. ASC supernatant had higher MSAF than plasma and tissue **A** and metastasis sites had influence on plasma **B**; ASC supernatant had higher actionable mutation rate than plasma in distant organ metastasis group **C** and only peritoneal metastasis group **D**

We retrospectively analyzed 304 GI cancer patients with peritoneal metastasis to further analyze the efficacy of ASC supernatants from patients for gene profiling. Targeted NGS of 1021 or 59 cancer-related genes was performed on 398 specimens for genomic profiling. First, we compared the MSAF in the different samples. MSAF was significantly higher in the ASC supernatant (50.00%) than in the plasma ctDNA (3.00%, *p* < 0.0001) and tissue samples (23.10%, *p* < 0.0001) (Fig. 1A), regardless of disease status (Supplementary Fig. 1). The ASC supernatant (100%, 45/45) had a higher frequency of somatic alterations detected than plasma (88.3%, 166/188) and tissues (98.7%, 154/156). Among the 304 patients, 169 had distant organ metastasis and 125 had only peritoneal metastasis. To explore the impact of the metastatic site on MSAF, we compared the MSAF of different samples from patients with distant organ metastasis to those from patients with peritoneal metastasis only. The MSAF of the plasma in patients with peritoneal metastasis was significantly lower than in patients with distant organ metastasis (1.00% vs. 5.60%, *p* < 0.0001); however, the MSAF was similar between the ASC supernatant (51.80% vs 50.80%, *p* = 0.70) and tissue samples (24.20% vs 22.90%, *p* = 0.33) from the same sets of patients (Fig. 1B). The actionable mutation rates in plasma, ASC supernatants, and tissue were 55.86%, 83.33%, and 75.29% in the distant organ metastasis group and 41.67%, 61.54%, and 64.29% in the peritoneal metastasis group, respectively. The ASC supernatant had a higher actionable mutation rate than plasma in both groups (Fig. 1C & 1D). There was a significant difference in distant organ metastasis between the two groups (83.33% vs. 55.86%, *p* = 0.01) (Fig. 1C). The ASC supernatants had a higher or comparable actionable mutation rate than those of the tissue samples.

### Paired ASC supernatant and ctDNA samples from 26 patients

**Fig. 2:**
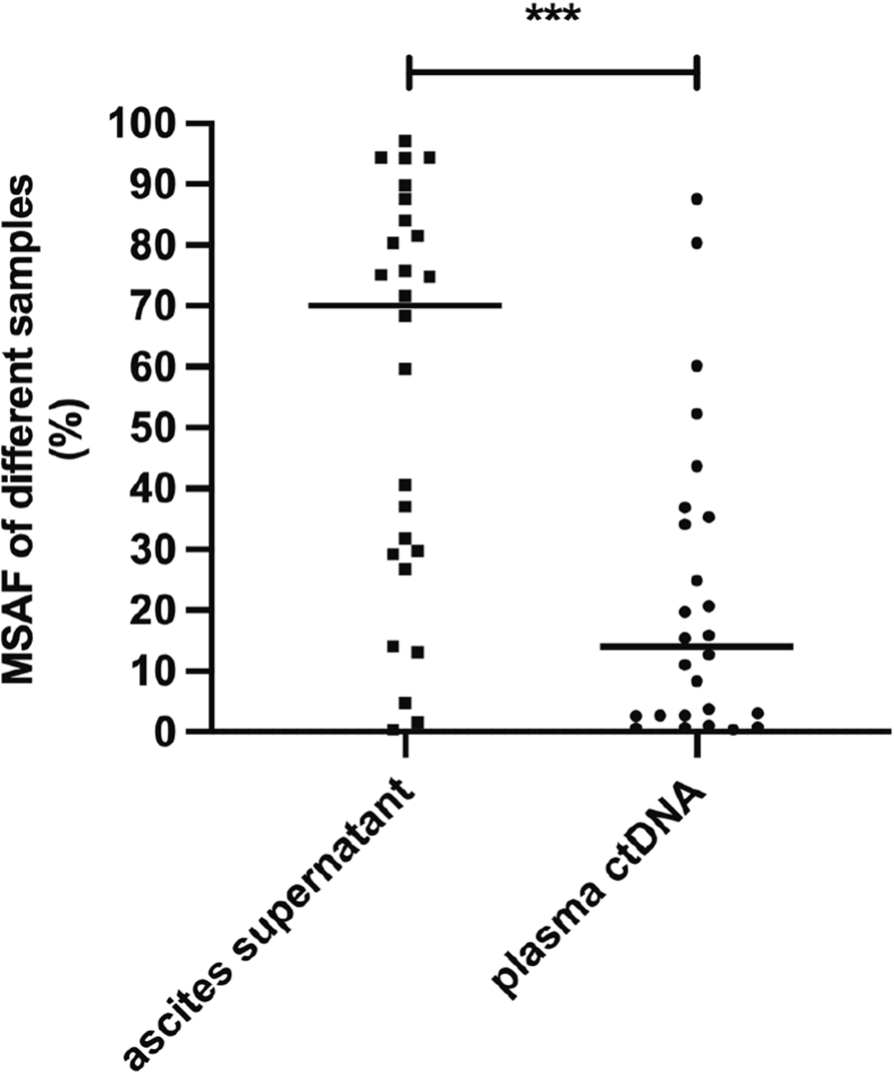
MSAF in ASC supernatant and plasma in 26 paired samples

Twenty-six patients had more than one sample, including 26 paired ASC supernatants and plasma ctDNA, 7 tissues, and 1 PE supernatant. All the samples had detectable somatic alterations. In the different subtype groups, 69.2% (18/26) of ASC supernatant samples, 50% (13/26) of plasma ctDNA samples, 57.1% (4/7) of tissues, and 100% (1/1) of PE supernatant samples had detectable actionable alterations (Table 2). The ASC supernatant had a higher detectable rate of actionable alterations, although there was no statistically significant difference. In the paired ASC supernatant and plasma ctDNA samples, seven patients had actionable alterations detected only in the ASC supernatant, and two patients had actionable alterations detected only in plasma ctDNA. The ASC supernatant accounts for more actionable alterations than plasma ctDNA. One CRC patient (P23) had a paired ASC supernatant and PE supernatant during disease recurrence, and the actionable alterations in the two samples had high consistency. Six patients had no distant organ metastasis, and three patients (P01, P09, and P10) had the same discovery in actionable alterations. However, actionable alterations were more frequently detected in the ASC supernatant than in the plasma ctDNA in the other three patients (P14, P15, and P20). We then compared the MSAF of the ASC supernatant and plasma ctDNA in the 26 patients. The MSAF in the ASC supernatant was significantly higher than that in the plasma ctDNA (70.00% vs. 14.10%, *p* = 0.0003) (Fig. 2). We speculate that the higher MSAF may explain the superior detection efficacy of the ASC supernatant compared to the plasma ctDNA, especially in patients with no distant organ metastasis.

**Table 2:**
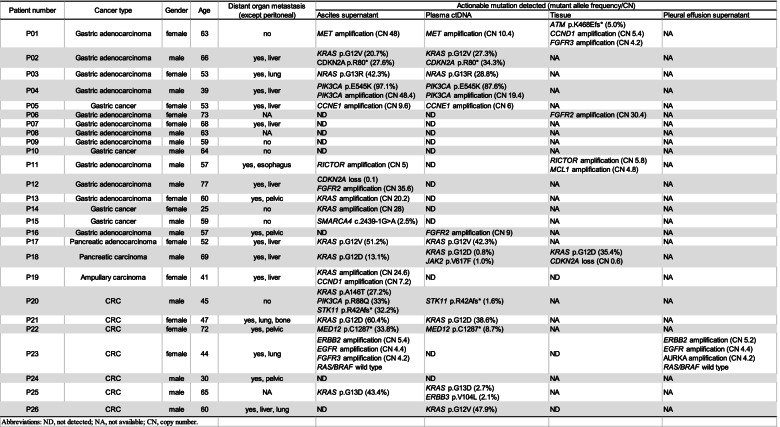
Actionable mutations detected in different specimens among 26 patients with paired samples

*Abbreviations: ND* not detected, *NA* not available, *CN* copy number

### Study design and lung cancer patient characteristics

This study enrolled 407 lung patients with pleural metastasis at diagnosis or during disease progression (Table 3). The median age at diagnosis was 60 (range 28–94), and 202 (49.6%) were female. The major histological subtype was adenocarcinoma (84.8%). One hundred and twenty-four (30.5%) patients had only pleural metastasis with stage M1a disease and 227 patients had the other organ metastases with stage M1b/c disease. Three hundred and twenty-one (78.9%) patients had previous systemic treatment. A total of 1002 specimens were collected to analyze the efficacy of mutation detection among actual different patient samples, including 389 plasma samples, 122 tissues, 446 PE supernatants, and 45 PE sediments.

**Table 3:** Clinical pathological characteristics of lung cancer patients

Characteristics	Patients (N=407)
Age, years	
median	60
range	28-94
Gender (n)	
female	202
male	205
Smoking history (n)	
Yes	105
No	173
NA	129
Histology subtype (n)	
Adenocarcinoma	345
Squamous	9
Adenosquamous	3
NA	50
M stage (n)	
M1a	124
M1b/c	227
NA	56
Previous system treatment (n)^#^	
yes	321
no	222
NA	35
Specimen (n)	
plasma ctDNA	389
tissue	122
pleural effusion supernatant	446
pleural effusion sediment	45

^#^Including 136 patients had multiple samples before and after system treatment

*Abbreviations: NA* not available, *ctDNA* circulating tumor DNA

### PE supernatant from real patients

**Fig. 3:**
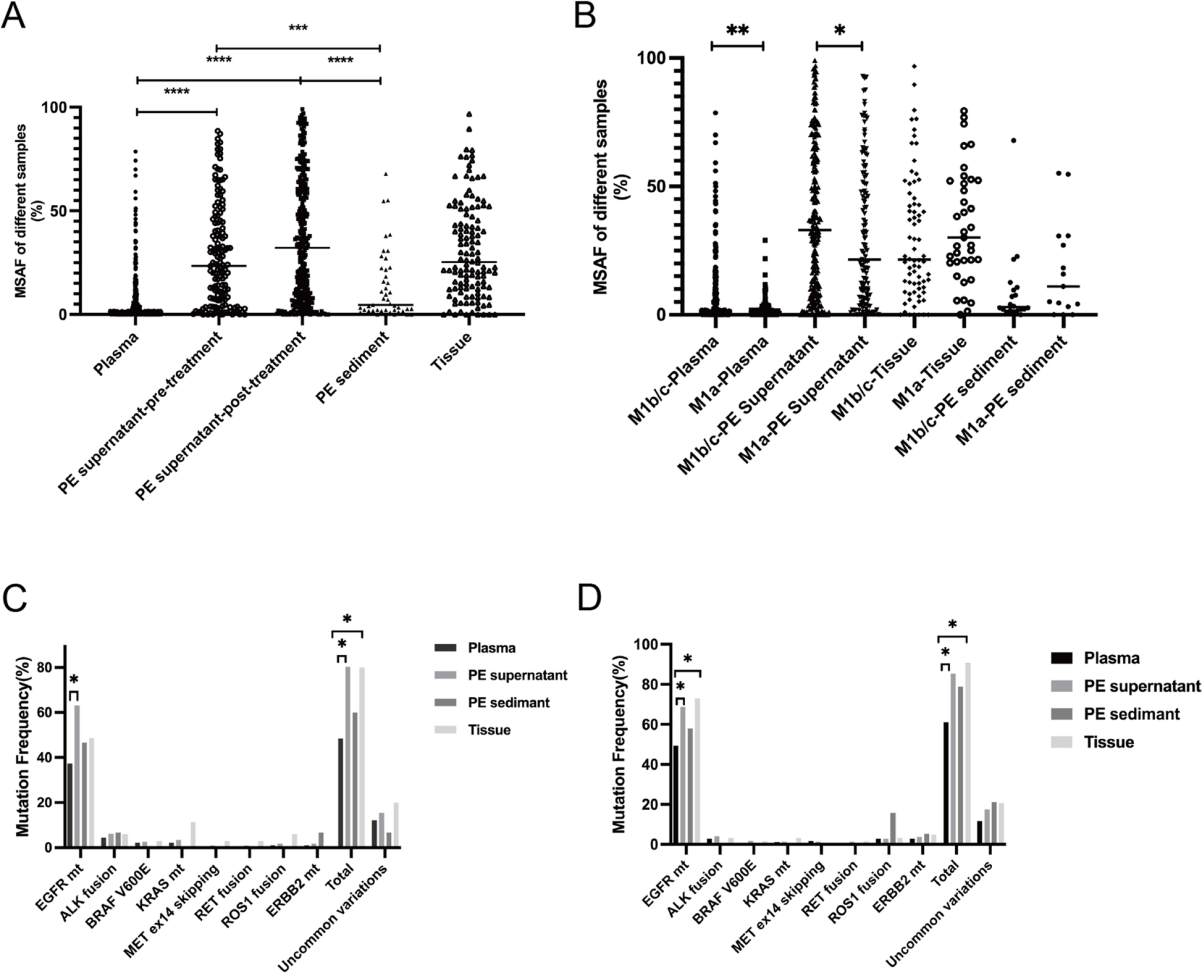
The mutation detection ability of different samples in lung cancer. PE supernatant had higher MSAF than plasma, sediment **A** and metastasis sites had influence on plasma and PE supernatant **B**; PE supernatant had higher driver mutation rate than plasma in stage M1a group **C** and stage M1b/c group **D**

We retrospectively analyzed 407 lung cancer patients with pleural metastasis to further analyze the efficacy of PE supernatant in actual patients. Targeted NGS of 1021 or 59 cancer-related genes was applied to 1002 specimens for genomic profiling. First, we compared the MSAF of different samples. The PE supernatant MSAF, regardless of treatment status (pre-treatment 23.45%, post-treatment 32.20%), was higher than plasma ctDNA (1.30%, *p* < 0.0001) and PE sediment (4.6%, *p* < 0.0001), and comparable to tissues (25.30%, *p* = 0.88) (Fig. 3A). Treatment had no influence on MSAF in the 49 paired PE supernatants (Supplementary Fig. 2). Plasma MSAF was higher in M1b/c patients than in M1a patients (1.60% vs. 1.05%, *p* = 0.0034) (Fig. 3B). PE supernatant MSAF was also higher in M1b/c than in M1a patients too (33.00% vs. 21.50%, *p* = 0.04). Tissue MSAF and PE sediment MSAF were comparable in patients with stage M1b/c and M1a disease, respectively.

Sequentially, we evaluated the targetable variations detected ability in different samples, including *EGFR*, *ALK, BRAF* V600E, *KRAS*, *MET* ex14 skipping, *RET*, *ROS1*, *ERBB2*, which have high evidence in lung cancer. Among stage M1a patients, PE supernatant, PE sediment, and tissue had higher total actionable mutation rates than that of plasma, especially the in PE supernatant (80.3% vs. 48.4%, *p* < 0.05) and tissue (80.0% vs. 48.4%, *p* < 0.05) (Fig. 3C). *EGFR* had a higher mutation rate in the other three samples than in plasma, especially in the PE supernatant (37.4% vs. 63.2%, *p* < 0.05). These results were also observed in patients with stage M1b/c disease (Fig. 3D). The mutation-detection ability of the PE supernatant was comparable to that of the tissue. *EGFR* uncommon mutation, *BRAF* V600E, *MET* ex14 skipping, *RET*, *ROS1*, and *ERBB2* were classified as uncommon variations. The PE supernatant had a higher frequency of uncommon variations than that of plasma, regardless of distant organ metastasis status (Fig. 3C & 3D).

### Paired plasma and PE supernatant samples in 139 patients

**Fig. 4:**
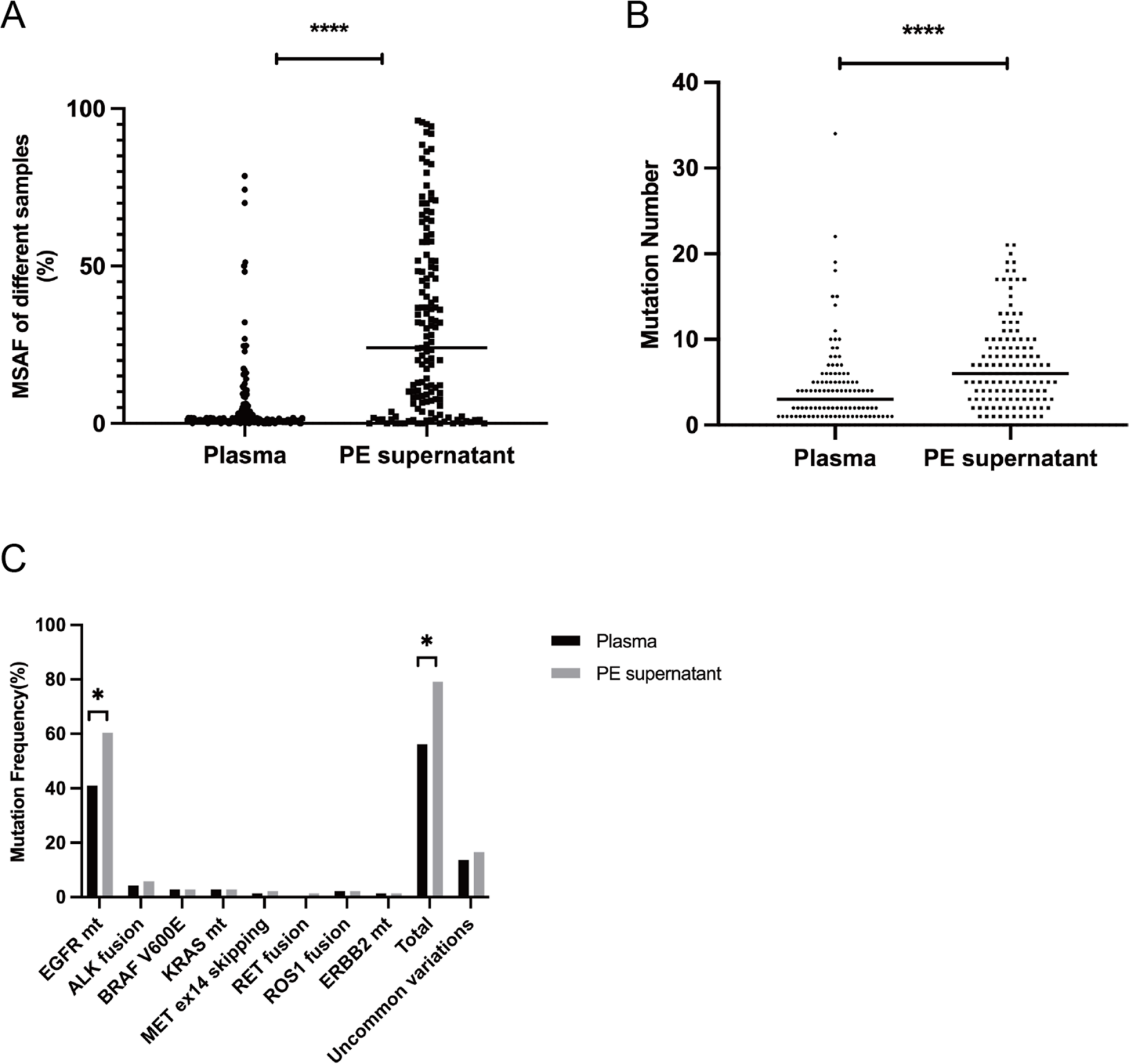
The mutation detection ability of PE supernatant and plasma in 139 lung cancer patients with paired samples. PE supernatant had higher MSAF **A** and more mutations **B** than plasma. PE supernatant had higher driver mutation detected rate than plasma **C**

One hundred and thirty-nine patients had simultaneously paired plasma and PE supernatant samples. Approximately 89.2% (124) of the plasma samples and 93.5% (130) of the PE supernatant samples had mutations. The PE supernatant had a higher MSAF (24.00% vs. 1.20%, *p* < 0.0001) (Fig. 4A) and more mutations were detected (6 vs. 3, *p* < 0.0001) (Fig. 4B) than that of plasma ctDNA. The PE supernatant had a higher detectable rate of targetable alterations than that of the plasma (79.1% vs. 56.1%, *p* < 0.05) (Fig. 4C, Supplementary Table 2); notably, there was a significant difference in *EGFR* mutation (Fig. 4C). The PE supernatant also had a higher frequency of uncommon variations than plasma (Fig. 4C). Representative cases demonstrate the clinical significance of these uncommon variants. In case 1, the *EZR-ROS1* positive lung adenocarcinoma patient who had multiple site metastases responded to crizotinib, an ROS1 inhibitor approved for *ROS1*-positive non-small cell lung cancer. In case 2, the *EGFR* ex20 P772_H773insYNP mutant lung adenocarcinoma patient with pleural metastasis had disease control using furmonertinib, a third-generation EGFR inhibitor.

## Discussion

Approximately 10%-40% of patients with GC and CRC have peritoneal metastases [6, 16]. Approximately 15% of patients with lung cancer have pleural metastasis at the first presentation, and 50% of patients develop PE during the duration of the disease [17]. ASC and PE are the common manifestations of peritoneal metastasis of digestive system tumors and pleural metastasis of lung cancer, respectively. Abdominal puncture and thoracentesis are widely performed in patients with these body fluids. The ASC and PE contents are generally relatively large, with an average of a few thousand milliliters. However, we have not seen much research on genomic profiling using these samples.

This study had a large sample size containing ASC, PE, tissue, and plasma samples from real patients. These results suggest that ASC and PE can provide important genomic information for different tumors. They can act as potential and effective samples used for molecular analysis when tumor tissues are unavailable. ASC and PE supernatants had higher or comparable MSAFthan that of tissue samples. Other reports have suggested that ASC and PE contain a larger amount of DNA for genomic analysis than that of compared to plasma [8, 9, 18]. In our study, we observed a significantly higher MSAF in ASC and PE supernatants than in plasma based on a large sample size. This indicates that ASC and PE had more abundant tumor-derived DNA. These results may be related to the sample characteristics. ASC and PE are local fluids in which the detected DNA may originate from necrotic or secreted all tumor cells, whereas tissue samples are taken from a part of the tumor cells, which may have heterogeneity. We also observed that MSAF in ASC and PE was not affected by treatment or disease status. These results indicate that ASC and PE are the most suitable sample types for clinical practice.

Targeted therapy has become an important clinical strategy, and matched therapies have provided longer survival [19, 20]. ASC and PE supernatants had a higher actionable mutation rates and more mutations detected than that of plasma in both GI and lung cancers. We observed that the ASC supernatant had more actionable mutations than that of plasma in 26 GI cancer patients with paired samples. We also observed this phenomenon in a limited number of paired ASC supernatant and tissues samples. In a CRC patient (P23) who had simultaneous ASC and PE, the actionable variations were highly consistent; however, no actionable mutations were detected in the plasma and tissue.

With the development of drugs and the advancements in detection technology, patients have more opportunities to receive targeted therapies. Oncogenic fusions, such as *ALK*, *NTRK1/3*, and *RET*, have been reported in GI cancer [21, 22]. These variations are of great significance in clinical treatment. CRC patients with oncogenic fusion can benefit from targeted therapy based on previous studies [23, 24]. We observed an *FGFR2* fusion from the ASC supernatant in gastric adenocarcinoma and a *RET* fusion in colon adenocarcinoma tissues. The FGFR inhibitors, erdafitinib and pemigatinib, have been approved for the treatment of *FGF-* fusion urothelial carcinoma and cholangiocarcinoma, respectively [25, 26]. The RET inhibitors, selpercatinib and pralsetinib have were also approved for the treatment of *RET*-fusion non-small cell lung cancer [27, 28]. We also observed a higher uncommon actionable mutation detection rate in the PE supernatant than in plasma in lung cancer.

The plasma had a lower MSAF and actionable mutation rate in patients who had only peritoneal or pleural metastasis than in those with widespread matastasis. The mutation detection ability of ASC and PE supernatant was comparable regardless of the stage.

This study had some limitations. First, this study was a retrospective study involving real-world patients without a rigorous research design; therefore, we only performed a preliminary exploration. Second, there are large differences in sample sizes among different sample types. For example, there are only 45 ASC samples; thus, the conclusion obtained in this study requires more samples to be added for further analysis. Third, many mutations were detected in the samples but lacked clinical efficacy data; these mutations need more attention and should be further studied.

## Conclusion

In a conclusion, cfDNA from ASC and PE supernatants provides more information for tumor genomic profiling than does cfDNA from plasma and PE sediment. ASC and PE supernatants have more advantages, regardless of distant organ metastasis status. Thus, ASC and PE supernatants could be better alternatives when tumor tissues are not available in the real-world setting, especially in patients with only peritoneal or pleural metastases.

## Supplementary Information

**Additional file 1:** 

**Additional file 2:** 

**Additional file 3:** 

**Additional file 4:** **Supplementary Table 1.** Gene list of 59 gene panel (A) and 1021 gene panel (B).

**Additional file 5:** **Supplementary Table 2.** Actionable mutations detected in different specimens among 139 lung cancer patients.

## Data Availability

The data and materials used and analyzed in the current study are available from the corresponding author on request.

## Abbreviations

ASC: Ascites
PE: Pleural effusion
NGS: Next generation sequencing
CRC: Colorectal cancer
GC: Gastric cancer
MSAF: Maximum somatic allele frequency
cfDNA: Cell-free DNA
SNV: Single nucleotide variants
InDel: Insertions and deletions
CNV: Copy number variations
