# Supplementary figures and images for: Liquid biopsy using ascitic fluid and pleural effusion supernatants for genomic profiling in gastrointestinal and lung cancers
Source: BMC Cancer. 2022 Sep 27;22:1020. doi: 10.1186/s12885-022-09922-5 (PMC9513868; doi:10.1186/s12885-022-09922-5)

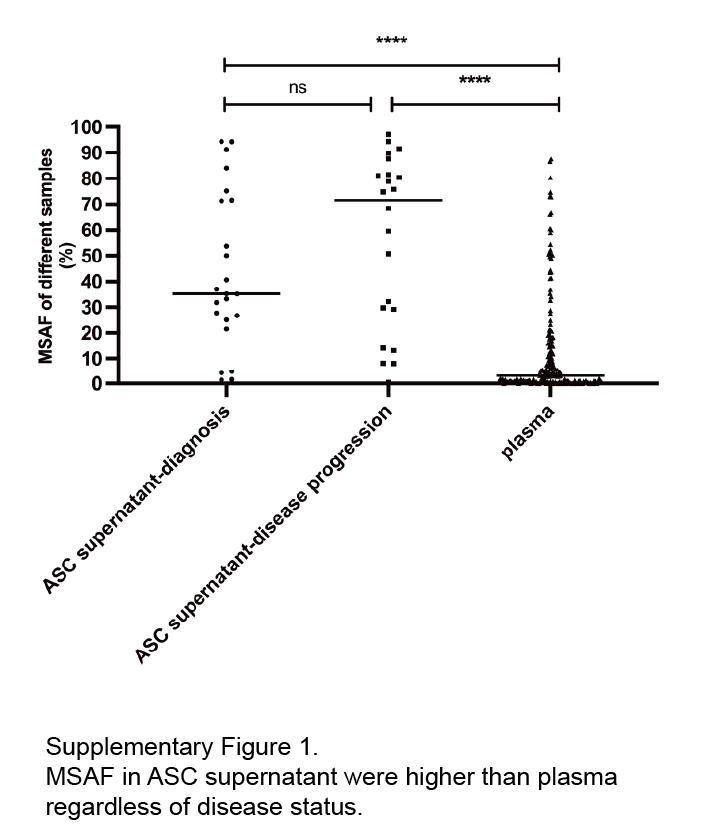

Supplement: Supplementary file 1 — Additional file 1: [file 12885_2022_9922_MOESM1_ESM.png]

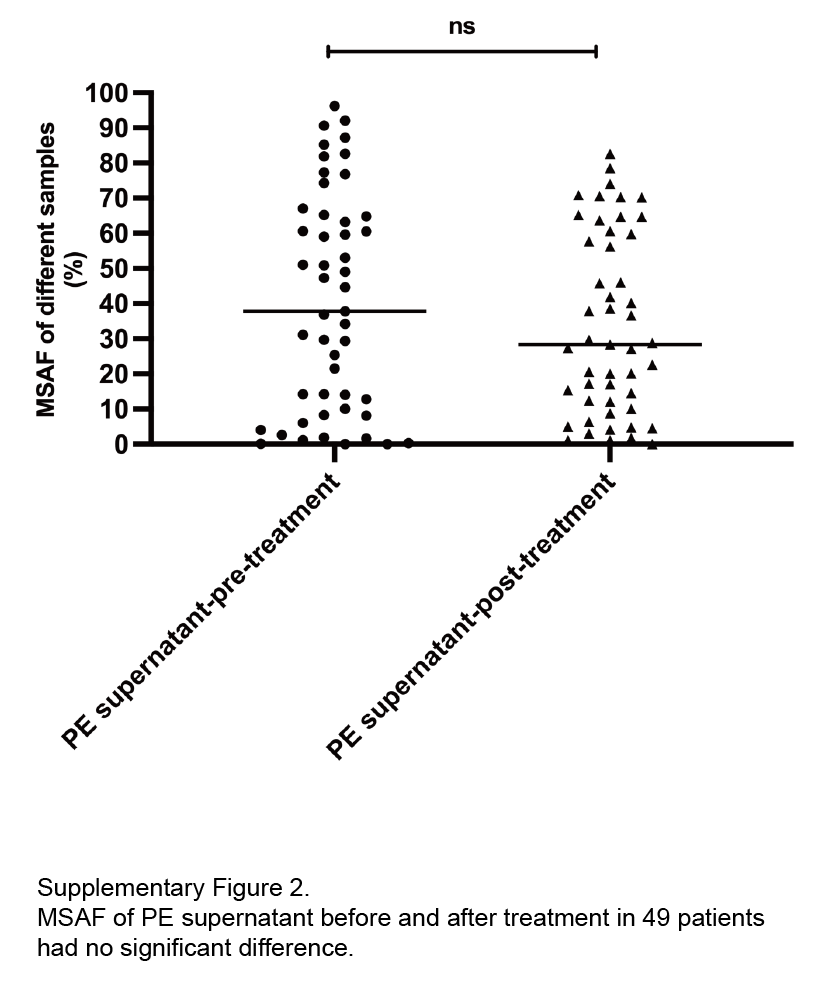

Supplement: Supplementary file 2 — Additional file 2: [file 12885_2022_9922_MOESM2_ESM.png]

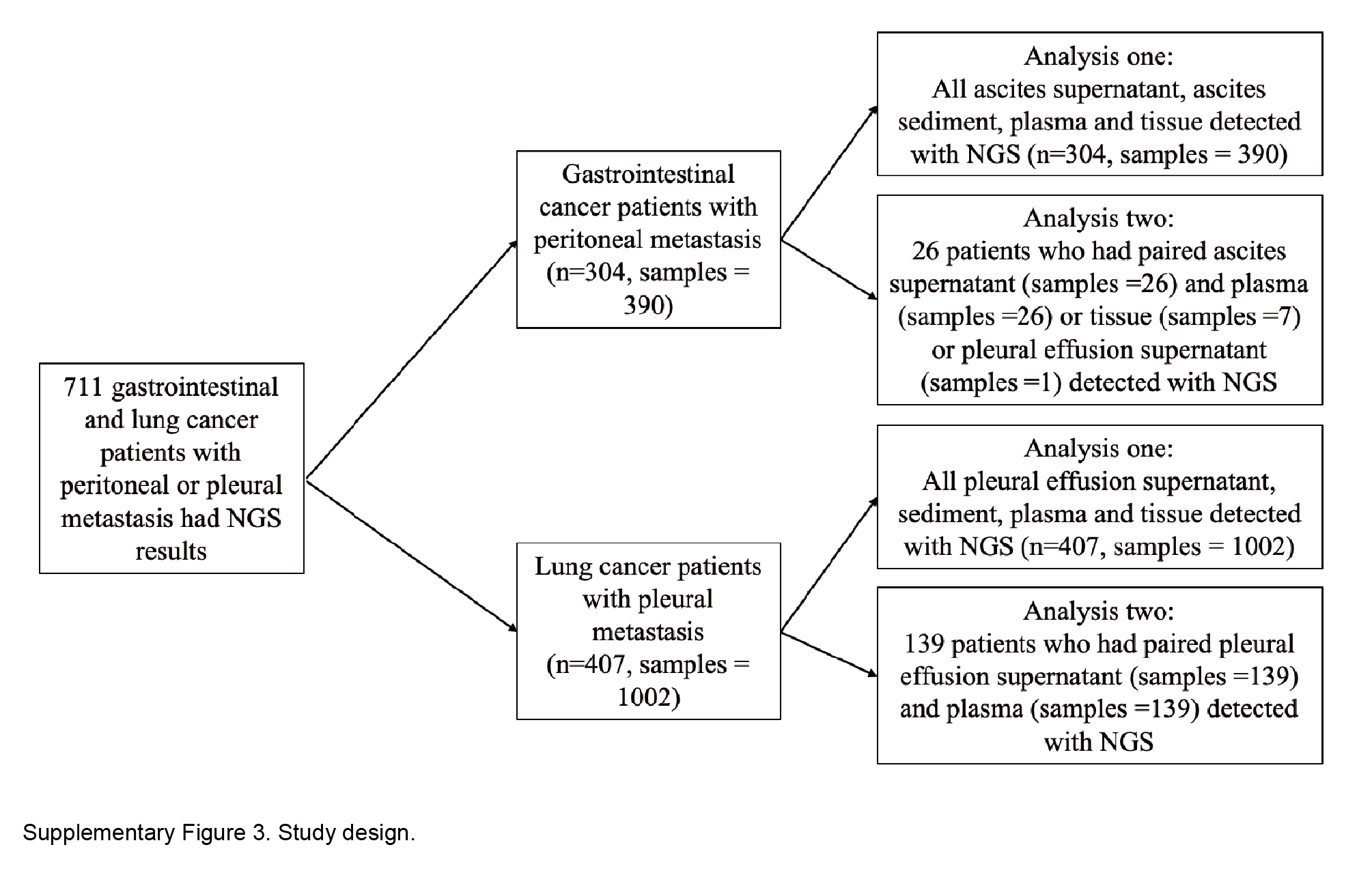

Supplement: Supplementary file 3 — Additional file 3: [file 12885_2022_9922_MOESM3_ESM.png]
